# Formation of vesicular structures from fatty acids formed under simulated volcanic hydrothermal conditions

**DOI:** 10.1038/s41598-023-42552-w

**Published:** 2023-09-14

**Authors:** Thomas Geisberger, Philippe Diederich, Christoph J. O. Kaiser, Kilian Vogele, Alexander Ruf, Christian Seitz, Friedrich Simmel, Wolfgang Eisenreich, Philippe Schmitt-Kopplin, Claudia Huber

**Affiliations:** 1 Structural Membrane Biochemistry, Technical University of Munich, BNMRZ, Lichtenbergstr. 4, 85748 Garching, Germany; 2 Research Unit Analytical BioGeoChemistry, Helmholtz Center Munich, 85764 Neuherberg, Germany; 3 Division for Electron Microscopy, Technical University of Munich, Lichtenbergstr. 4, 85748 Garching, Germany; 4 Physics of Synthetic Biological Systems, Physics Department E14, Technical University of Munich, Am Coulombwall 4a, 85748 Garching, Germany; 5 Faculty of Physics, LMU Munich, Schellingstraße 4, 80799 Munich, Germany; 6 Excellence Cluster ORIGINS, Boltzmannstraße 2, 85748 Garching, Germany

**Keywords:** Biochemistry, Biogeochemistry

## Abstract

Microscopic compartmentalization is beneficial in synthetic chemistry and indispensable for the evolution of life to separate a reactive “inside” from a hydrolyzing “outside”. Here, we show compartmentalization in aqueous solution containing mixtures of fatty acids up to 19 carbon atoms which were synthesized by one-pot reactions of acetylene and carbon monoxide in contact with nickel sulfide at 105 °C, reaction requirements which are compatible to Hadean Early Earth conditions. Based on confocal, dynamic light scattering (DLS) and transmission electron microscopy (TEM) measurements, vesicle-like structures with diameters of 10–150 nm are formed after solvent extraction and resolubilisation. Moreover fluorescent dye was encapsulated into the structures proving their vesicular properties. This self-assembly could also have occurred on Early Earth as a crucial step in establishing simple membranes of proto-cells as a prerequisite in the evolution of metabolism and life.

## Introduction

Compartmentalization and the formation of protocells are indispensable factors for the evolution of early life. Only by this principle, the formation of complex molecular entities including catalytic polymers is enabled as a prerequisite for the emergence of self-replicating systems in the evolution of life. In present biochemistry the membranes of eukaryotes and bacteria are bilayers of phospholipids mostly consisting of phosphoglycerides or glycolipids^1^, which separate and protect a well-organized cell inside from the outside environment. In archaea, isoprenyl ether derivatives are used for membrane formation^2^. In early abiotic Earth scenarios, simpler amphiphiles like fatty acids may be considered as candidates for vesicle formation and therefore precursors for protocell membranes^3,4^.

Self-assembly of vesicles was described as an essential step in the evolution of the first membrane like structures of protocells^5–8^ and has been thoroughly investigated for octanoic acid^9^, decanoic acid^10,11^ and longer chain fatty acids^9,12–14^. While for pure fatty acids, the critical conctentration for vesicle formation (CVC) is fairly high, for mixtures of different chain length, the CVC often is significantly lower^15,16^. Likewise, the presence of alcohols lowers the CVC significantly^17^. Lipid formation from formic acid or oxalic acid and self-assembly of glyceryl alkanoates was shown under simulated hydrothermal conditions^18,19^. Furthermore fatty acids up to a chain length of 12 carbon atoms were extracted with organic solvents from Murchison carbonaceous meteorite^20,21^ and these extracts were shown to have the capacity to form vesicles if reconstituted in water^22^.

In living organisms fatty acids are formed from acetyl-CoA. In analogy to this C2 chain elongation acetylene was used as primordial carbon source^23^. In Reppe-like reactions^24^ carbon monoxide is used as additional carbon source for the formation of the carboxylic acid group and as reducing agent for stepwise reduction of the first formed unsaturated carboxylic acids. Concerning the primordial accessibility of the required starting materials acetylene and CO would have been available in prebiotic Hadean volcanic-hydrothermal fluid flows^25–28^ and acetylene was as well shown to be present on Jupiter ^29^ and Titan ^30^. Catalytic transition metal sulfides like NiS catalysts would have been exposed in the mineral walls of hydrothermal flow ducts^31^.

By using established procedures^12–17,22,32^ we here show, that mixtures of mainly unsaturated fatty acids formed from acetylene and carbon monoxide in the presence of NiS are able to form vesicular structures. These were examined by dynamic light scattering (DLS) measurements, negative stain transmission electron microscopy (TEM) and fluorescence measurements after dye inclusion.

## Results

### Extended formation of fatty acids under hydrothermal conditions

**Figure 1:**
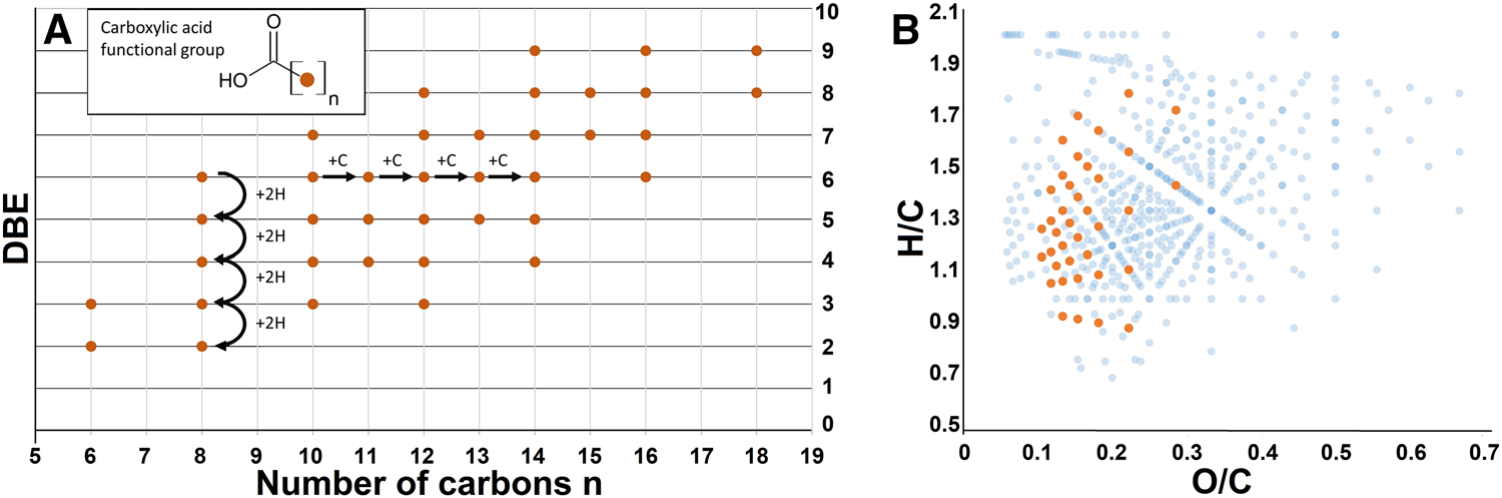
Unsaturated fatty acids detected by negative ionization mode electrospray FT-ICR-MS. (**A**) Double bond equivalent (DBE) representing the amount of unsaturation compared to the number of carbons attached to the carboxylic acid group. (**B**) Van Krevelen plot showing the overall complexity of the CHO-space including various functionalizations.

A mixture of short chain fatty acids H–[CH_2_–CH_2_]_x_(CH=CH)_y_]–COOH (x + y ≤ 4) was detected previously by GC/MS in experiments using acetylene (C_2_H_2_), carbon monoxide (CO) and nickel sulfide (NiS) under anoxic conditions at 105 °C ^23^. Now, by extending the analytical toolset to high field fourier-transform ion cyclotron resonance mass spectrometry (FT-ICR-MS) analysis we are able to detect a mixture of fatty acids up to a chain length of 19 carbon atoms. Assigned signals reflect mainly unsaturated carboxylic acids with odd numbered carbon chains. Functionality assignment of the elemental compositions was done through comparison with ^13^C-labelled carbon monoxide experiments. Only elemental compositions carrying a single carbon label and fitting the elemental composition of unsubstituted fatty acids were considered (Fig. 1A). In addition, we detected carbon-labelled even numbered carboxylic acid formulas and signals with elemental compositions in line with potential keto acids, hydroxy acids and dicarboxylic acids which can be calculated from the highly precise m/z values (+ /− 0.3 ppm error at 250 m/z) (Fig. 1B).

The overall extent of molecular complexity in the vesicle-forming mixture can be visualized in a van Krevelen plot (Fig. 1B). Formulas are arranged according to their hydrogen/carbon and oxygen/carbon ratios, resulting in a visual categorization of different saturation and oxidation levels. Starting from GC–MS confirmed molecules, hypothetical connecting lines suggest the class of fatty acids.

### Behavior of the fatty acids mixture

**Figure 2:**
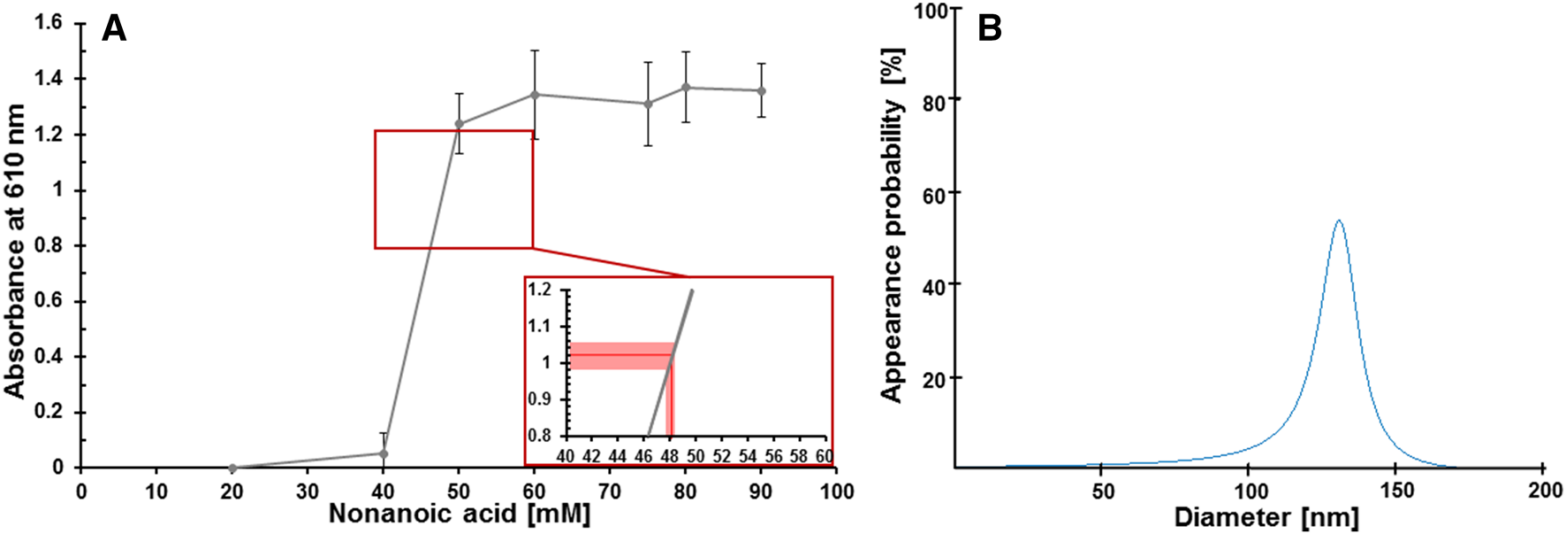
Determination of the concentration and aggregation state of extracted fatty acid mixtures. (**A**) Concentration determination by the pinacyanol colorimetric assay. Defined concentrations of nonanoic acid were used for calibration in two independent measurements in bicine buffer (grey). The inset illustrates the concentration determination for the extracted fatty acid mixtures: Averaged absorbance value (horizontal red line) from two independent reaction mixtures with standard deviation is shown with the corresponding average concentration (vertical red line). The steep increase in the absorbance at 610 nm indicates the concentration range at which the fatty acids form aggregates. (**B**) Mean size distribution of the particels measured by DLS of four replicates from reaction mixtures containing acetylene, CO and NiS after solvent extraction, drying and rehydration in water. Size distribution calculated from the DLS data results in a mean size of about 135 nm for the particles.

As shown by the van Krevelen plot the reaction mixture from acetylene and CO forms a large number of different fatty acids and fatty acid-like structures, with only low concentrations for each single fatty acid species. Therefore, a colorimetric assay with pinacyanol chloride can be deployed^15,33^ to estimate the overall fatty acid concentration and the aggregation status of the fatty acid mixture. The absorbance at 610 nm increases explicitly with the formation of aggregates in the solution, which can also be visually observed by a chromatic shift from purple to blue. Nonanoic acid is used for calibration and exhibits aggregation above a concentration of about 40 mM. To determine the fatty acid concentration in our reaction mixtures, here interpreted as nonanoic acid equivalents, the reaction mixture is extracted with chloroform and resolved in bicine solution achieving a fivefold concentration of the fatty acid components. The so treated fatty acid mixtures from two independent reaction setups show an absorbance at 610 nm corresponding to about 48 mM in the “nonanoic acid scale” (Fig. 2A).

### Formation of vesicular structure

Encouraged by these findings the reaction mixtures were further investigated using established protocols^17^. After solvent extraction, drying and rehydration in water the mixture is measured by GC–MS, dynamic light scattering (DLS) and transmissions electron microscopy (TEM). This procedure again implies a fivefold concentration of the fatty acids compared to the original solution. GC–MS analysis of this extract confirms unsaturated fatty acids with eleven carbon atoms (Fig S1). DLS measurement was performed with four replicates showing a reproducible signal (Fig. S2). The correlation function of the DLS is used to determine the diameter of the particles with a CONTIN-like algorithm. These data can be described by a Weibull extremal probability distribution, which results in a mean diameter of 135 nm and a dispersion value (sample standard deviation) of 15 nm (Fig. 2B).

**Figure 3:**
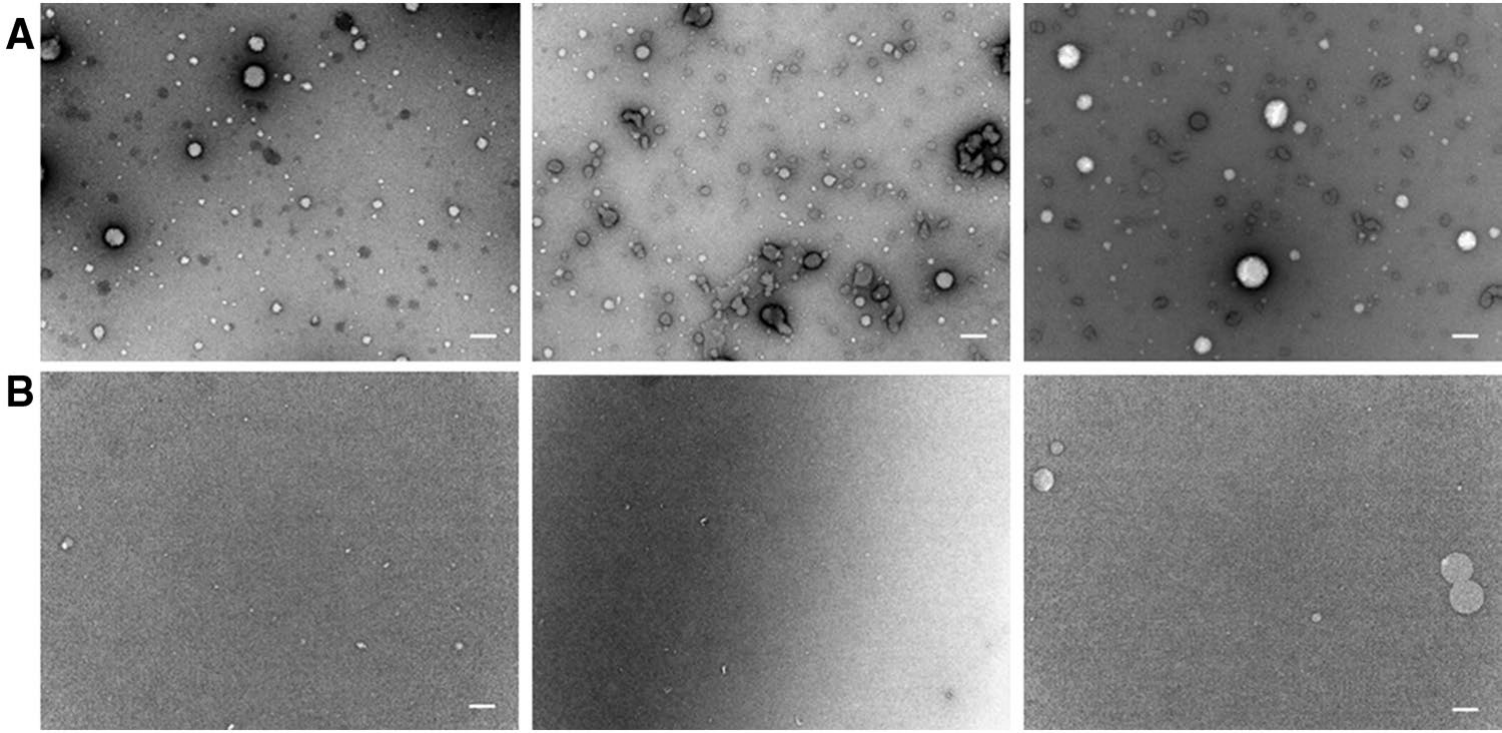
Negative-stain TEM micrographs of extracted fatty acids in aqueous solution. (**A**) Spherical structures obtained from fatty acid forming reaction mixtures containing acetylene, CO and NiS after solvent extraction, drying and resolubilisation in water. (**B**) TEM micrographs from identical experiments to A with argon instead of acetylene and CO, a setup which shows no fatty acid formation. The scale bar represents 100 nm in all micrographs.

In negative-stain TEM micrographs, the samples of the reaction mixtures containing acetylene, CO and NiS after solvent extraction, drying and rehydration show circular (10 to 150 nm) and rarely elongated structures (Fig. 3A). These structures are absent, when argon is substituted for acetylene and CO in a control reaction (Fig. 3B).

As shown earlier, NiS catalyzed experiments with acetylene and CO produce mainly unsaturated straight chain fatty acids with a chain length C5-C9 in a concentration range of 10 mM^23^. Solvent extraction followed by drying and silylation of the reaction products now reveals GC–MS signals with typical m/z values of unsaturated carboxylic acids up to 11 carbon atoms (Fig. S2) and FT-ICR MS indicates fatty acids up to a chain length of C19 (Fig. 1).

**Figure 4:**
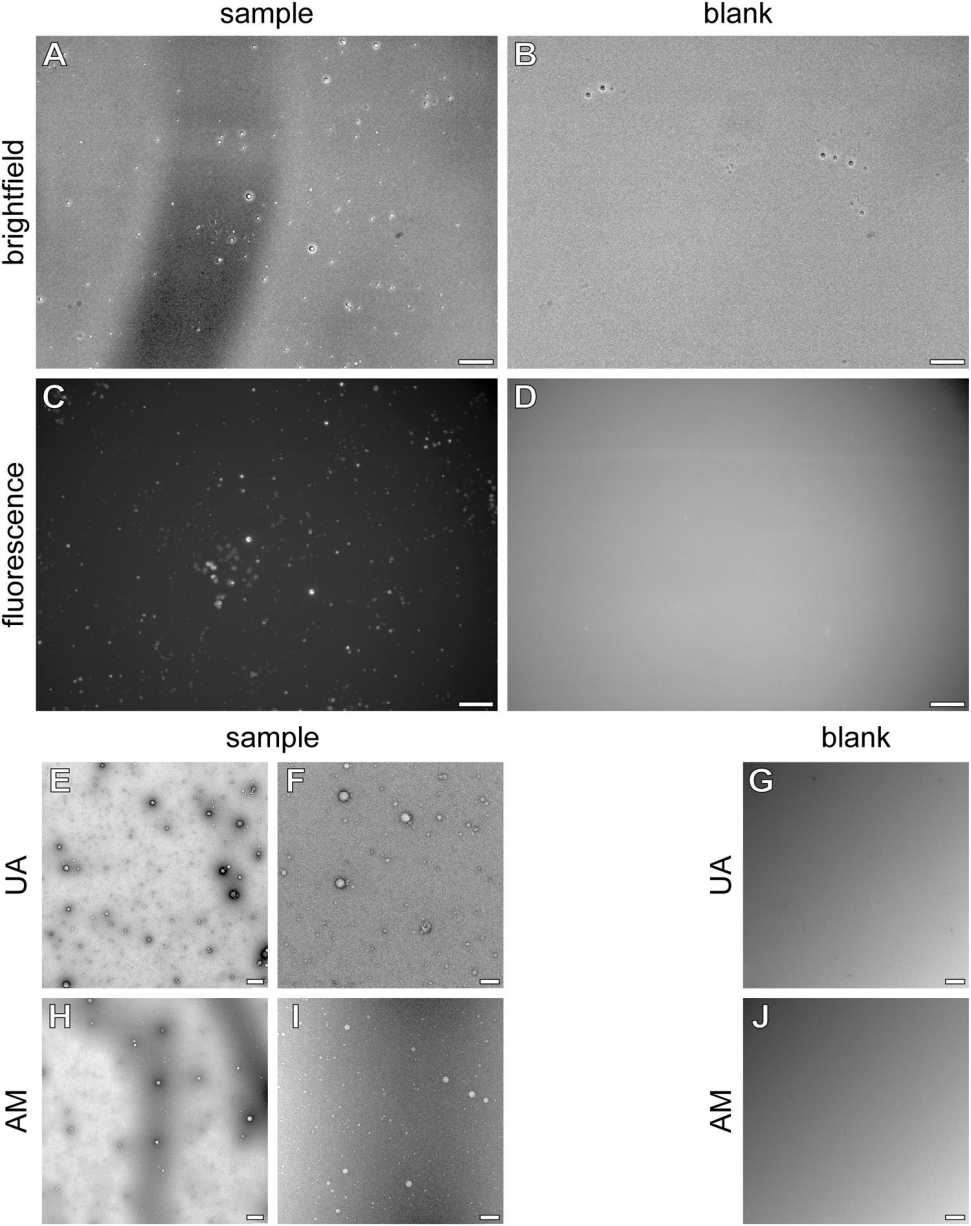
Characterization of vesicles. Confocal micrographs of a vesicle sample (**A**, **C**) and a control sample (**B**, **D**) formed by hydration of the chloroform extracted fatty acid mixture in 100 mM POPSO buffer (pH 8.2) in the presence of 10 µM pyranine. Maximum projections of the brightfield (**A**, **B**) and green fluorescence (**C**, **D**) channels are shown. Free chromophore was removed by passing the vesicle solution over a sepharose G50 spin column before microscopy. The scale bar represents 10 µm in panels (**A**–**D**). Electron micrographs of the raw rehydrated vesicle suspension shown in panels A and B before chromophore removal. (**E**, **F**) Negative stain (2% uranyl acetate). (**H**, **I**) Negative stain (2% ammonium molybdate, pH 8.4). Panels E and H were recorded at 5000 × nominal magnification, the scale bar represents 1 µm. Panels F, I, G, J were recorded at 29,000 × nominal magnification, the scale bar represents 100 nm.

After only roughly fivefold concentration through extraction and resolubilisation in water spherical structures are formed. DLS and TEM measurements both show structures with diameters of about 100 nm (Figs. 2 and 3). The slight difference in sizes shown by the different methods can be explained by the fact that DLS from suspensions of particles with varying size is strongly influenced by larger particles, since they scatter more light. Therefore, when larger particles are present in the suspension, even at a low quantity, the detection of smaller events becomes problematic^34^. The aqueous solution had a pH value of about 3.5. At this pH value oil droplet formation has also to be considered instead of vesicle formation. To assure vesicle formation the procedure was repeated using 0.1 M POPSO buffer pH 8.2, which is lowered to pH 7.9 through the rehydration of the fatty acid mixture. At this pH value only vesicles are to be expected^35^. The vesicular structures were observed by confocal microscopy (brightfield; Fig. 4A) and by TEM using both, uranyl acetate (UA; Fig. 4E and F) and ammonium molybdate (AM; Fig. 4H and I) as negative stain. For the unequivocal proof of vesicle formation we could show that the so formed vesicles are able to encapsulate a fluorescent dye like pyranine (Fig. 4C). Before microscopy free chromophore was removed by passing the vesicle solution over a sepharose G50 spin column. The vesicular structures vary in size, as would be expected for vesicles from a mixture of different fatty acids and show diameters up to about 150 nm (Fig. 4), a typical vesicle size as shown in^16,35^. The diameter of micelles would be limited by the fatty acid chain length: they typically range below 5 nm for e.g. dodecylchains^36^. In reaction setups using argon instead of acetylene and CO under otherwise identical treatment the vesicular structures are not detected (Fig. 4B,D,G and J).

## Discussion

The origin of cellular life on Earth required the evolution of membrane-like structures. The simultaneous emergence of biomolecules through prebiotic chemistry in aqueous medium may have led to encapsulated “reaction vessels”, which are capable of protecting hydrolysis sensitive molecules (e.g. peptides and nucleotides) from the influence of excess water.

**Figure 5:**
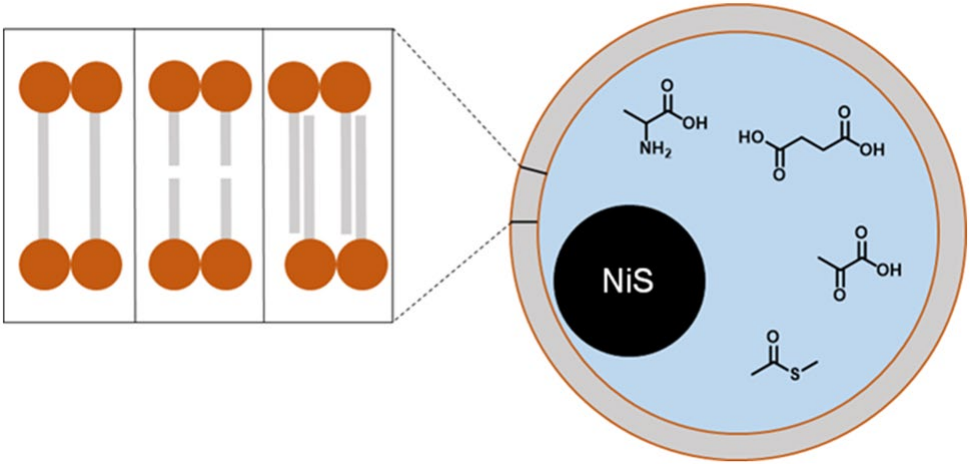
Hypothetical detached lipid vesicle with internal NiS-nanoparticle; black: catalytic NiS-nanoparticle; blue: enclosed aqueous phase with metabolic intermediates; red: polar groups of the carboxylic acids; grey: apolar tails of the carboxylic acids (monolayer, bilayer or interdigitated form).

In a volcanic hydrothermal flow channel consecutive steps would lead to precursors of first protocells^37^:

1. Surface metabolism: organic molecules are built on catalytic transition metal sulfides from inorganic volcanic gases. A primitive metabolic cycle is formed from these organic components^38^
2. Surface lipophilization: fatty acids or isoprenoid acids are amongst these organic components^23^. Accumulation of theses primitive lipids leads to a lipophilisation of the catalytic mineral substructure with reduction of water activity and therefore a promotion of condensation reactions, e.g. important for peptide formation^39,40^.
3. Membrane formation: with increasing surface concentration of the lipids an inversion will take place and still surface-bonded membranes (interdigitated or bilayers) are formed^37^.
4. Detached lipid vesicles with internal NiS-nanoparticle: the growing surface-bonded lipid membranes will produce semicellular structures with a “cytosolic” metabolism on enclosed mineral nano-particles (Fig. 5).

The concentration of the fatty acid mixture formed under 1 bar reaction pressure acetylene and CO maybe too low for spontaneous vesicle formation, but a higher reaction pressure as usual at submarine volcanic hydrothermal vents would increase the yields of these fatty acids. Also conceivable is the subsequent concentration of fatty acids in capillary flow ducts or in thermal gradients^41^. Fatty acids are thermostable and chemically robust molecules, which would live long enough for such concentration scenarios, and additionally a complex mixture of fatty acids shows a dramatically lowered critical vesicle concentration compared to the single fatty acid^15^. Additionally, it was shown that in combination with alcohols^17^, terpenoids^16^, interaction with mineral surfaces^42^ and salinity^16^ significantly lowered vesicle forming concentrations are observed. In volcanic hydrothermal vents, we would expect all of these conditions to be met and mixtures to be present, and consequently the formation of vesicles would probably be promoted even at relatively low concentrations.

Our experiments indicate the possible self-assembly of vesicle like structures from fatty acids formed from acetylene and CO under hydrothermal volcanic conditions. The concomitant formation of important biomolecules under these conditions could lead to a possible encapsulation of reaction mixtures and therefore be a pathway to first protocells on Earth.

## Methods

All chemicals were purchased from Sigma-Aldrich GmbH (D-Steinheim) in the highest purity available. Acetylene was purchased from Linde AG (D-Pullach). Carbon monoxide and Argon 4.6 were purchased from Westfalen AG (D-Münster).

### Fatty acid formation

In a typical run a 125 ml glass serum bottle was charged with 1.0 mmol NiSO_4_·6H_2_O and closed with a silicon stopper. Three times the bottle was evacuated and filled with argon, finally ending in a deaerated state. Subsequently the bottle was filled with argon-saturated water (calculated for the end volume of 5 ml), with 1.0 mL argon-saturated 1 M Na_2_S solution, with 1.0 mL 1 M NaOH solution and finally with 60 ml CO and 60 ml acetylene, using gas-tight syringes for the injections. Reactions were carried out at 105 °C. After 7 days the reaction mixture was allowed to cool down. Formation of fatty acids was controlled by GC–MS measurements as described earlier (Scheidler et al. 2016). In a blank run with otherwise identical composition acetylene and CO were replaced by argon. No formation of fatty acids and vesicle like structures was observed in this run.

### FT-ICR mass spectrometry

Samples were taken from the serum bottle with a syringe and centrifuged for 5 min at 15,000 rpm. 100 µl of the supernatant were diluted in 900 µl methanol and centrifuged again to remove the precipitated salt. 70 µl of the centrifuged sample were then diluted again in 930 µl methanol to obtain the final sample concentration.

Analysis was performed on a high-field Fourier Transform Ion Cyclotron Resonance mass spectrometer from Bruker Daltonics—Solarix with a 12 T magnet from Magnex. The mass spectra were acquired with a 4 megaword (MW) time domain. The system was calibrated with L-Arginine clusters in negative ionization mode (5 mg L^−1^ L-Arginine solved in methanol). For each sample, scans were accumulated in the mass range of 122–1000 amu. Ions were accumulated for 300 ms. The pressure in the hexapole was 3 × 10^−6^ mbar, and the pressure in the ICR vacuum chamber was 6 × 10^−6^ mbar. An Apollo ii (Bruker Daltonics) ESI source was used. The supernatant was directly injected via a microliter pump system (flowrate: 120 µl h − 1).

### Pinacyanol assay

Nonanoic acid equivalents were used as calibration standards. Nonanoic acid equivalents were pipetted into a pointed flask according to the respective concentrations (20, 40, 50, 60, 75, 80, 90 µM). To the samples, 1 mL of bicine solution (0.2 M, pH 7.4) was added and treated for 5 min in an ultrasonic bath. The samples were transferred to cuvettes, mixed with 100 µL of aqueous pinacyanol solution (250 µM) and incubated for 15 min at room temperature. The absorption of the solution was then measured at 610 nm (Photometer Novaspec II, Pharmacia Biotech; New Jersey US). Each concentration was prepared twice and the mean values were used as calibration curve.

To determine the corresponding concentration of nonanoic acid equivalents in the reaction setup an entire content of a serum bottle (5 mL) was centrifuged at 4000 rpm for 10 min. The aqueous supernatant was transferred to a separatory funnel and acidified with HCl (1 M). The solution was extracted three times with 20 mL chloroform. The solid residue was treated in the same way. The organic phases were combined and dried with Na_2_SO_4_ (anhydrous). The solvent was evaporated on a rotary evaporator and resolved in 1 ml bicine solution as described above. The absorption measurement at 610 nm was performed identical as for the nonanoic acid calibration equivalents.

### Fatty acid extraction and preparation for DLS and TEM

1 ml of the aqueous reaction mixture was subjected to a centrifugation step at 7.500 × *g* for 7 min. The supernatant was transferred to a 4 ml glass vial. 100 µl of 6 M HCl was added to acidify the mixture, then 1 ml of CHCl_3_ was added. The mixture was vortexed for 2 min. After phase separation the organic phase (CHCl_3_) was transferred to a new vial. The aqueous phase was extracted two more times. The combined organic phases were dried with Na_2_SO_4_. The CHCl_3_ phase was transferred in to a round bottom flask and the solvent was removed with a rotary evaporater. The fatty acids will form a layer on the glass. To break up the layer and form vesicles, 200 µl of H_2_O or POPSO/NaOH pH 8.2 were added and the flask was sonificated for 15 min or spun overnight at ambient temperature, respectively. The resulting liquid mixtures were used for DLS and TEM measurements as described in the results section.

### GC–MS measurement of fatty acids from the organic extract

Sample preparation was performed as described for the pinacyanol assay. The dried residue was dissolved in 250 μl anhydrous acetonitrile and derivatized with 250 μl

N-tert-butyldimethylsilyl-N-methyltrifluoroacetamide for 30 min at 70 °C.

Analysis was performed with GC–MS, using GC-2010 coupled with MS-QP2010 Ultra (Shimadzu GmbH, D-Duisburg) with a 30 m × 0.25 mm × 0.25 μm fused silica capillary column (Equity TM5, Supelco, Bellefonte, PA, USA) and AOC-20i auto injector. Temperature program and settings:

0–3 min at 150 °C; 3–73 min at 150–220 °C, 1 °C/min; 73–81 min at 220–300 °C, 10 °C/min 81–83 min at 300 °C; injector temperature: 260 °C; detector temperature: 260 °C; column flow rate: 1 mL/min; scan interval: 0.5 s; injection volume 1.0 μl.

### Dynamic light scattering

The DLS measurements were performed with a DynaPro Nanostar (Wyatt technology corporation) with disposable covets to minimize cross-contamination. For a measurement 50 µL sample volume was used. The correlation function was averaged over 50 single measurements, each 2 s long. The data were further processed with the DYNAMICS software using a CONTIN-like algorithm to deduce a full size distribution of the particles.

### Transmission electron microscopy

Copper grids with carbon film (~ 20 nm) were prepared in-house. Before sample application, the grids were treated in a glow-discharge apparatus (air plasma) for 30 s to increase hydrophilicity. The sample was applied and allowed to adsorb for 60 s and excess solution was blotted off. A solution of 2% uranyl acetate or 2% ammonium molybdate/ammonia pH 8.0 in water was applied and incubated for 30 s on the grid before the stain was blotted off. The sample was allowed to air-dry before being imaged in a JEM1400Plus (Jeol, Akishima, Japan) transmission electron microscope at a magnification of 30,000 × or on a FEI (Hillsboro, Oregon, USA) Tecnai F20 at magnifications 5000 × or 29,000x.

### Confocal and fluorescence microscopy

For fluorescence microscopy, samples were reconstituted in 100 mM POPSO/NaOH pH 8.2 with 10 µM pyranine. Free label was removed by passing the samples over sepharose G50 spin colums (Cytiva, Marlborough, Massachusetts, USA) pre-equilibrated with POPSO/NaOH pH 8.2. Two thin parallel strings of silicone grease were placed onto an object slide and a coverslip was placed on these to yield a cleft for sample mounting. The cleft was filled by pipetting the sample onto the slide and exploiting capillary force for filling. The sample was sealed with silicone grease to prevent evaporation. The sample was examined on a Corrsight microscope (FEI, Groningen, Netherlands) using the spinning disk andromeda detector for green channel fluorescence and bright field imaging. Stacks were recorded with a Z-slice spacing at the Nyquist spacing and at positions close to the coverslip-liquid interface.

### Supplementary Information

Supplementary Figures.

## Data Availability

All data are available in the main text or the supplementary materials.
