# Supplementary material for: Formation of vesicular structures from fatty acids formed under simulated volcanic hydrothermal conditions
Source: Sci Rep. 2023 Sep 14;13:15227. doi: 10.1038/s41598-023-42552-w (PMC10502091; doi:10.1038/s41598-023-42552-w)
Supplement: Supplementary file 1 — Supplementary Figures. [file 41598_2023_42552_MOESM1_ESM.pdf]

Supplementary Materials for

Formation of vesicular structures from fatty acids formed under simulated volcanic hydrothermal conditions

Thomas Geisberger, Philippe Diederich, Christoph Kaiser, Kilian Vogele, Alexander Ruf, Christian Seitz, Friedrich Simmel, Wolfgang Eisenreich, Philippe Schmitt-Kopplin, Claudia Huber*

Correspondence to:claudia.huber@tum.de

This PDF file includes:

Figs. S1 to S2

Fig. S1.

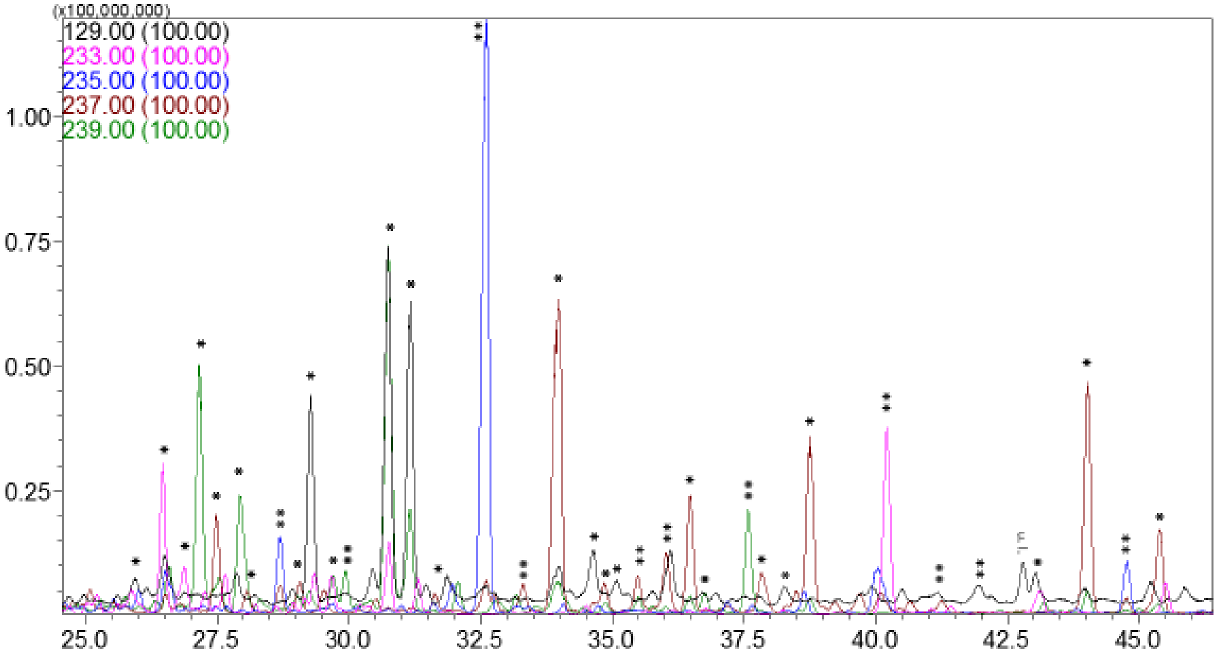

Fig S1: GC/MS mass traces from the chloroform extract of the reaction products; selected for unsaturated fatty acids with 11 carbon atoms. Mass traces are defined as follows:
m/z 129: [C5H9O2Si]+ • A typical fragment of silylated carboxylic acids
m/z 233: [C13H17O2Si]+ • A typical fragment of silylated C11:5 fatty acids
m/z 235: [C13H19O2Si]+ • A typical fragment of silylated C11:4 fatty acids
m/z 237: [C13H21O2Si]+ • A typical fragment of silylated C11:3 fatty acids
m/z 239: [C13H23O2Si]+ • A typical fragment of silylated C11:2 fatty acids
Fatty acids C11:1 and C11:0 were not detected
*: defines one carbon label from ^{13}CO
**: defines two carbon labels from ^{13}CO
u.l.: defines no carbon label from ^{13}CO

Double labeling indicates dicarboxylic acids or keto acids

Fig. S2.

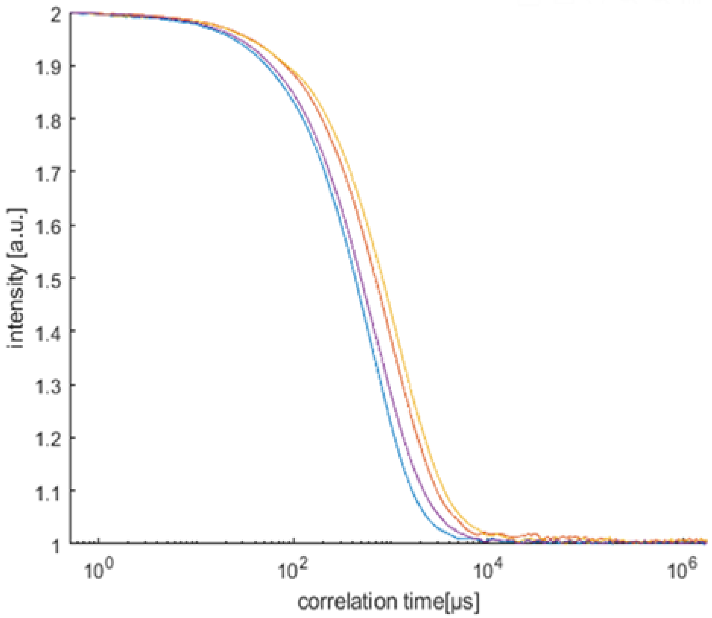

Fig S2: DLS measurement with four independent replicates of reaction mixtures containing acetylene, CO and NiS after solvent extraction, drying and rehydration in water. Size distribution calculated from DLS correlation time is in a range of about 130 nm.
